# Glomerular filtration rate and albuminuria predict mortality independently from coronary artery calcified plaque in the Diabetes Heart Study

**DOI:** 10.1186/1475-2840-12-68

**Published:** 2013-04-18

**Authors:** Amanda J Cox, Fang-Chi Hsu, J Jeffrey Carr, Barry I Freedman, Donald W Bowden

**Affiliations:** 1 Center for Human Genomics, Wake Forest School of Medicine, Winston-Salem, NC, USA; 2 Center for Diabetes Research, Wake Forest School of Medicine, Winston-Salem, NC, USA; 3 Department of Biochemistry, Wake Forest School of Medicine, Winston-Salem, NC, USA; 4 Department of Biostatistical Sciences, Wake Forest School of Medicine, Winston-Salem, NC, USA; 5 Department of Radiologic Sciences, Wake Forest School of Medicine, Winston-Salem, NC, USA; 6 Department of Internal Medicine - Nephrology, Wake Forest School of Medicine, Winston-Salem, NC, USA

**Keywords:** Coronary artery calcified plaque, Mortality, Albuminuria, Type 2 diabetes, Risk prediction

## Abstract

**Background:**

Risk stratification in individuals with type 2 diabetes (T2D) remains an important priority in the management of associated morbidity and mortality, including from cardiovascular disease (CVD). The current investigation examined whether estimated glomerular filtration rate (eGFR) and urine albumin:creatinine ratio (UACR) were independent predictors of CVD-mortality in European Americans (EAs) with T2D after accounting for subclinical CVD.

**Methods:**

The family-based Diabetes Heart Study (DHS) cohort (n=1,220) had baseline measures of serum creatinine, eGFR, UACR and coronary artery calcified plaque (CAC) assessed by non-contrast computed tomography scan. Cox proportional hazards regression was performed to determine risk for all-cause mortality and CVD-mortality associated with indices of kidney disease after accounting for traditional CVD risk factors and CAC as a measure of subclinical CVD.

**Results:**

Participants were followed for 8.2±2.6 years (mean±SD) during which time 247 (20.9%) were deceased, 107 (9.1%) from CVD. Univariate analyses revealed positive associations between serum creatinine (HR:1.56; 95% CI:1.37–1.80; p<0.0001) and UACR (1.59; 1.43–1.77; p>0.0001) and negative associations between serum albumin (0.74; 0.65–0.84; p<0.0001) and eGFR (0.66; 0.58–0.76; p<0.0001) with all-cause mortality. Associations remained significant after adjustment for traditional CVD risk factors, as well as for CAC. Similar trends were noted when predicting risk for CVD-mortality.

**Conclusions:**

The DHS reveals that kidney function and albuminuria are independent risk factors for all-cause mortality and CVD-mortality in EAs with T2D, even after accounting for CAC.

## Background

Cardiovascular disease (CVD) remains a serious complication in individuals with type 2 diabetes (T2D), accounting for greater than 60% of all-cause mortality [1]. However, not all individuals with T2D experience the same risk for macrovascular disease and other complications [2]; this variable risk likely reflects different underlying environmental and inherited risk factors, diabetes duration, extent of subclinical CVD, and clinical management. As such, risk stratification remains an important priority in the prevention of T2D-associated morbidity and mortality.

The Diabetes Heart Study (DHS) is a family-based study enriched for T2D affected individuals. Recent findings from the DHS have shown that coronary artery calcified plaque (CAC) determined by non-invasive, non-contrast computed tomography (CT) scanning and accepted as reflecting subclinical CVD, is an independent predictor of both all-cause mortality [3] and CVD-mortality [4]. Observed odds ratios for CAC scores exceeding 1000 were 6.7–11.2 [3]. In an effort to further refine these models, we have investigated whether other non-invasive clinical measures were independent predictors of mortality in T2D-affected individuals after accounting for CAC.

Reduction in kidney function (or estimated glomerular filtration rate; eGFR) is an established risk factor for adverse CVD outcomes in a range of settings, including acute coronary syndrome [5], heart failure [6,7] and acute myocardial infarction [8]. However, uncertainty remains as to direct causality and the mechanisms underpinning relationships between kidney function and CVD [9,10]. In addition, although a range of renal function indices are associated with CVD-mortality in both population-based studies [11,12] and T2D-affected cohorts [13-15], adjustment for underlying vascular disease burden is frequently overlooked. We have previously reported strong independent associations between albuminuria and CAC in the DHS [16]. Likewise, microalbuminuria has also been associated with increased presence and progression of CAC in the Multi-Ethnic Study of Atherosclerosis [17] as well as with increased carotid intima-media thickness, another measure of subclinical CVD [18]. Given the potential interplay between kidney function, endothelial dysfunction, atherosclerotic plaque formation and CVD outcomes, this investigation examined whether measures of kidney function and albuminuria were predictive of mortality in European Americans (EAs) with T2D, independent of CAC.

## Methods

### Study design and sample

The DHS cohort includes 1,220 self-described EA individuals from 475 families. Briefly, the DHS recruited siblings concordant for T2D without advanced renal insufficiency, manifesting as serum creatinine concentration >2 mg/dL, or end-stage renal disease. When possible, one non-T2D affected sibling was also recruited. T2D was clinically defined as diabetes developing after the age of 35 years and actively treated with insulin and/or oral agents, in the absence of historical evidence of ketoacidosis. Diagnoses were confirmed by baseline measurement of fasting blood glucose and glycosylated hemoglobin (HbA_1C_). Full ascertainment and recruitment criteria have been previously described in detail [19,20].

Study protocols were approved by the Institutional Review Board at Wake Forest School of Medicine, and all participants provided written informed consent prior to participation. Participant examinations were conducted in the Clinical Research Unit of Wake Forest Baptist Medical Center, and included interviews for medical history (including self-reported history of prior CVD events or intervention) and health behaviors, anthropometric measures, resting blood pressure, electrocardiography, fasting blood sampling for laboratory analyses including fasting glucose, HbA_1C_, lipids, serum albumin and creatinine concentration. Estimated GFR was calculated using the 4-variable Modification of Diet in Renal Disease (MDRD) equation [21]. A spot urine collection was obtained for determination of urine albumin: creatinine ratio (UACR).

Coronary artery calcified plaque (CAC) was measured using fast-gated helical CT scanners, with calcium scores calculated as previously described [22,23]. CAC is widely accepted as reflecting the burden of subclinical CVD.

### Mortality

Vital status was determined for all participants from the National Social Security Death Index maintained by the United States Social Security Administration. For participants confirmed as deceased, length of follow-up was determined from the date of the initial study visit to date of death. For deceased participants, copies of death certificates were obtained from relevant county Vital Records Offices to confirm cause of death. For all other participants the length of follow-up was determined from the date of the initial study visit to the end of 2011. Cause of death was categorized based on information contained in death certificates as CVD-related (myocardial infarction, congestive heart failure, cardiac arrhythmia, sudden cardiac death, peripheral vascular disease, and stroke), cancer, infection, end-stage renal disease, accidental, or other (including obstructive pulmonary disease, pulmonary fibrosis, liver failure and Alzheimer’s dementia).

### Statistical analysis

Summary statistics were calculated including means and standard deviations (SD), medians and ranges for continuous variables, and count and percentages for categorical variables. Continuous variables were transformed prior to analysis to approximate normality. To evaluate the association of serum albumin, serum creatinine, UACR and eGFR with all-cause mortality and CVD-mortality a survival analysis was used. Variables reflecting kidney disease and serum albumin were considered as both ordinal (Q1-4 derived from increasing quartile ranges) and continuous variables. In order to compare the relative importance, kidney disease (continuous) variables were standardized for analysis of associations with outcome.

Curves of cumulative incidence of both all-cause mortality and CVD-mortality for increasing quartiles of each of the kidney disease measures were plotted for exploratory analyses (Additional file 1: Figures S1-S4). Due to the inclusion of related individuals in the DHS, Cox proportional hazards models with sandwich-based variance estimation were used to examine the relationships between measures of kidney disease and both all-cause mortality and CVD-mortality. Initially an exploratory test for trend across increasing quartiles of the measures of kidney disease was performed to examine relationships with all-cause and CVD-mortality. Analyses of the simple univariate associations using continuous variables were then performed. Each of these associations was subsequently adjusted for (i) age, sex, T2D affection status and use of angiotensin-converting enzyme (ACE) inhibitor and angiotensin-receptor blocker (ARB) medications (partially adjusted) and (ii) age, sex, T2D affection status, ACE/ARB medication use, body mass index (BMI), current smoking, hypertension, dyslipidemia, and self-reported history of prior CVD (fully adjusted). To assess whether measures of kidney disease predicted mortality independently of subclinical CVD the fully adjusted models were further adjusted for CAC.

Receiver operating characteristic (ROC) curves were computed for models containing traditional CVD risk factors (as used in fully adjusted models above) and with addition of either a measure of kidney disease, CAC, or both kidney disease measures and CAC. The areas under the curves were used to assess the ability of measures of kidney disease to predict all-cause mortality or CVD-mortality after adjusting for traditional CVD risk-factors and CAC. The difference in area under the curve between two models was tested using Delong’s method [24].

All analyses were performed using SAS version 9.2 (SAS Institute Inc., Cary, NC) with the exception of the ROC analyses which were performed using Stata software, version 12.1 (StataCorp, College Station, TX). Statistical significance was accepted at p<0.05.

## Results

Demographic and clinical characteristics of the DHS cohort are presented in Table 1. As anticipated in a T2D-enriched sample, a predominance of traditional CVD risk factors were evident including high BMI, hypertension, dyslipidemia and prior CVD events. Scores for CAC reflect a substantial burden of subclinical CVD in these diabetes-affected individuals and their siblings. Measurements of proteinuria and kidney function in the DHS revealed 27.9% with UACR >30 mg/g and 38.5% with eGFR<60ml/min/1.73m^2^. The cohort was followed for 8.2 ± 2.6 years (mean ± SD) during which time 247 (20.9%) participants were deceased, 107 (9.1%) from CVD causes.

**Table 1:** Demographic and clinical characteristics of European American Diabetes Heart Study (DHS) participants

	**Mean ± SD or %**	**Median (range)**
**Demographic information**		
Age (years)	62.0 ± 9.3	62.6 (35–86)
Gender (% female)	53.3%	
Type 2 diabetes affected (%)	83.7%	
Diabetes duration (years)	10.4 ± 7.2	8 (0–46)
% smoking (current or past)	58.7%	
Self-reported history of prior CVD (%)	39.0%	
Deceased (%)	20.9%	
Deceased from CVD (%)	9.0%	
**Body composition**		
Height (cm)	168.6 ± 9.7	168.4 (122.8–202.0)
Weight (kg)	90.6 ± 20.2	88.3 (40.0–209.2)
BMI (kg/m^2^)	31.8 ± 6.5	30.8 (16.6–58.0)
**Medications**		
Lipid lowering (%)	44.7%	
ACR/ARB (%)	44.7%	
**Blood pressure**		
Systolic BP (mmHg)	139 ± 19	138 (70–260)
Diastolic BP (mmHg)	73 ± 10	73 (37–106)
Hypertension (%)	84.9%	
**Blood biochemistry**		
Glucose (mg/dL)	139.3 ± 55.3	126 (16–463)
Hemoglobin A_1C_ (%)	7.3 ± 1.7	6.9 (3.5–18.3)
Total cholesterol (mg/ dL)	186.8 ± 42.3	183 (65–427)
HDL-cholesterol (mg/dL)	43.1 ± 12.4	41 (8–104)
LDL-cholesterol (mg/dL)	105.2 ± 32.6	103 (12–236)
Triglycerides (mg/dL)	200.9 ± 131.6	172 (30–1310)
Albumin (g/dL)	4.2 ± 0.3	4.2 (3.2–5.3)
**Kidney function**		
Serum creatinine (mg/dL)	1.08 ± 0.25	1.10 (0.40–1.75)
Estimate glomerular filtration rate (mL/min/1.73m^2^)	67.1 ± 20.2	64.3 (20.0–179.9)
Urine albumin/creatinine ratio (mg/g)	91.1 ± 319.9	12.4 (0.5–4165.0)
**Vascular imaging**		
Coronary artery calcified plaque (Agatston units)	1657 ± 3148	330 (0–50415)

Cumulative incidence for all-cause and CVD-mortality increased with increasing serum creatinine and UACR and decreasing serum albumin and eGFR (Additional file 1: Figures S1-S4). A formal test for trend using ordinal measures (derived from quartile cut-points) supported an overall trend for increasing creatinine (HR: 1.42; 95% CI: 1.26–1.60; p=2.74×10^-7^) and UACR (HR: 1.55; 1.37–1.74; p=1.18×10^-12^) and decreasing serum albumin (HR: 0.82; 95% CI: 0.73–0.92; p=0.0005) and eGFR (HR: 0.71; 0.63–0.81; p=1.07×10^-7^) to be associated with risk for all-cause mortality. Serum creatinine (HR: 1.55; 1.28–1.88; p=6.51×10^-6^), UACR (HR: 1.87; 1.55–2.26; p=1.10×10^-10^) and eGFR (HR: 0.69; 0.58–0.83; p=5.86×10^-5^) were similarly associated with risk for CVD-mortality. This trend was less prominent for serum albumin (HR: 0.85; 0.71–1.02; p=0.09). Similar patterns were noted when analyses were repeated including T2D affected individuals only (Additional file 1: Table S1).

Univariate analyses of continuous measures of kidney disease further supported the positive association of serum creatinine and UACR with all-cause and CVD-mortality (Table 2). Each SD increase in these measures was associated with ~1.5 fold (all-cause mortality) and ~1.75 fold (CVD-mortality) increase in risk, respectively. These positive associations remained significant after adjustment for traditional CVD risk factors including T2D affection status, BMI, current smoking, hypertension, dyslipidemia, and prior CVD (Table 2). Similarly, univariate analyses supported the negative associations of serum albumin and eGFR with mortality outcomes. Each SD increase in these measures was associated with a 25–35% (all-cause) and a 20–40% (CVD-mortality) reduction in risk respectively. These negative associations also remained significant after adjustment for traditional CVD risk factors (Table 2). Results were essentially unchanged when analyses were repeated including T2D affected individuals only (Additional file 1: Table S2).

**Table 2:** Association between continuous measures of kidney disease and mortality with covariate adjustment as indicated

	**Unadjusted**		Partially adjusted*		**Fully adjusted†**		**Fully adjusted† + CAC**	
	**HR (CI)**	**p-value**	**HR (CI)**	**p-value**	**HR (CI)**	**p-value**	**HR (CI)**	**p-value**
All-cause mortality								
Serum Albumin	0.74 (0.65–0.84)	7.13×10^-6^	0.72 (0.63–0.83)	3.80×10^-6^	0.71 (0.61–0.82)	4.02×10^-6^	0.71 (0.61–0.82)	6.81×10^-6^
Serum Creatinine	1.56 (1.37–1.80)	1.53×10^-10^	1.32 (1.12–1.54)	0.0007	1.28 (1.10–1.50)	0.002	1.29 (1.10–1.52)	0.002
UACR	1.59 (1.43–1.77)	1.48×10^-20^	1.48 (1.31–1.67)	1.66×10^-10^	1.41 (1.25–1.59)	3.95×10^-8^	1.37 (1.22–1.56)	3.83×10^-7^
eGFR	0.66 (0.58–0.76)	2.54×10^-9^	0.76 (0.65–0.89)	0.0006	0.77 (0.66–0.90)	0.001	0.77 (0.65–0.91)	0.002
CVD mortality								
Serum albumin	0.81 (0.66–0.99)	0.04	0.80 (0.65–0.98)	0.03	0.77 (0.62–0.95)	0.02	0.78 (0.63–0.98)	0.03
Serum creatinine	1.76 (1.42–2.19)	3.29×10^-7^	1.52 (1.19–1.96)	0.0009	1.44 (1.13–1.84)	0.003	1.48 (1.15–1.91)	0.003
UACR	1.79 (1.55–2.07)	4.00×10^-15^	1.65 (1.39–1.95)	4.74×10^-9^	1.54 (1.29–1.83)	1.32×10^-6^	1.50 (1.26–1.79)	5.22×10^-6^
eGFR	0.62 (0.50–0.77)	1.08×10^-5^	0.69 (0.54–0.88)	0.002	0.71 (0.56–0.91)	0.006	0.69 (0.53–0.89)	0.005

*UACR*=urine albumin:creatinine ratio; *eGFR*=estimated glomerular filtration rate.

*adjusted for age, sex, type 2 diabetes affection status, ACE/ARB medication use; † adjusted for age, sex, type 2 diabetes affection status, body mass index, current smoking, hypertension, dyslipidemia, ACE/ARB medication use, prior cardiovascular disease.

For each measure of kidney disease, fully adjusted models were further adjusted for CAC. Measures of proteinuria and kidney disease remained significantly associated with all-cause and CVD-mortality after additional adjustment (Table 2). Using quartile ranges to define high-risk individuals as those with serum creatinine and UACR values in the highest quartile and serum albumin and eGFR values in the lowest quartile of each respective distribution, these analyses revealed that high-risk individuals were at 1.4–1.8 fold increased risk for all-cause mortality (Table 3) and 1.9–2.4-fold increased risk for CVD-mortality (Table 3). Results were essentially unchanged when analyses were repeated including T2D affected individuals only (Additional file 1: Table S3).

**Table 3:** Association between categorical measures of kidney disease and all-cause mortality and cardiovascular disease (CVD) mortality

			**All-cause mortality**		**CVD-mortality**	
	**Quartiles**	**Quartlie ranges**	**HR (95% CI)**	**p-value**	**HR (95% CI)**	**p-value**
Serum albumin†	Q1-Q3	3.2–4.4	1.54 (1.14–2.08)	0.005	1.35 (0.84–2.17)	0.21
	Q4	4.4–5.3				
Serum creatinine*	Q1-Q3	0.4–1.2	1.36 (0.99–1.87)	0.06	2.02 (1.24–3.29)	0.005
	Q4	1.2–1.75				
UACR*	Q1-Q3	0.5–37.9	1.81 (1.38–2.39)	1.99×10^-5^	2.38 (1.61–3.53)	1.50×10^-5^
	Q4	37.9–4165.0				
eGFR†	Q1-Q3	20.0–75.0	1.58 (1.16–2.16)	0.004	1.94 (1.23–3.05)	0.004
	Q4	75.0–179.9				

*UACR*=urine albumin:creatinine ratio; *eGFR*=estimated glomerular filtration rate.

Associations based on proportional hazards regression models adjusted for age, sex, type 2 diabetes affection status, body mass index, current smoking, hypertension, dyslipidemia, ACE/ARB medication use, prior CVD and coronary artery calcified plaque.

*For serum creatinine and UACR, quartiles were established based on increasing values across the distribution such that quartile 4 (Q4) captures the highest risk individuals. †For serum albumin and eGFR, quartiles were established based on decreasing values across the distribution such that Q4 again captures the highest risk individuals.

Area under the curve analysis confirmed the utility of serum albumin in the prediction of all-cause mortality (AUC: 0.73 to 0.75; p=0.008) and of UACR in the prediction of CVD-mortality (AUC: 0.74 to 0.77; p=0.03) when included in models containing traditional risk factors. In contrast, serum creatinine and eGFR did not improve prediction beyond traditional risk factors (Additional file 1: Table S4). The tendency for serum albumin and UACR to improve prediction of all-cause (AUC: 0.73 to 0.78; p=0.02) and CVD-mortality (AUC: 0.74 to 0.80; p=0.06) respectively was still evident, although to a lesser extent in the models including CAC.

## Discussion

Cardiovascular complications and CVD-mortality are increased in individuals with T2D. As such, improved risk prediction may lead to reductions in morbidity and pre-mature mortality. We previously demonstrated that albuminuria was positively associated with CAC in the T2D-enriched DHS cohort [16] and that CAC powerfully predicted mortality in this sample [3,4]. We now extend these observations by demonstrating that proteinuria and eGFR remained predictive of all-cause mortality and CVD-mortality after accounting for the presence of traditional CVD risk factors and CAC. CAC is known to be associated with reduced eGFR and albuminuria. As such, it remained important to demonstrate that measures of kidney disease were independently associated with mortality even after subclinical CVD was accounted for in the analyses. To date, relatively few studies have assessed mortality after a mean eight year follow-up based on the independent effects of CAC and parameters of kidney disease.

That measures of kidney disease predicted mortality in a T2D-enriched sample is broadly consistent with the existing literature. A number of investigations have identified the associations of both albuminuria with CVD-mortality and eGFR with CVD-mortality, consistently demonstrating that these were independent associations [11-14,25,26]. Albuminuria and eGFR have also been confirmed to predict all-cause mortality in a recent large meta-analysis using general population cohorts of varying ethnicities [27]. An additional meta-analysis utilizing cohorts with multiple underlying CVD risk factors reported that both UACR and eGFR predicted mortality independent from traditional CVD risk factors [26]. Although some of these investigations accounted for traditional CVD risk factors and history of prior CVD, the present DHS analysis demonstrates that measures of kidney function and UACR remained independently associated with all-cause mortality and CVD-mortality after accounting for traditional CVD risk factors and CAC [4]. This was a critical analysis due to the relationships between UACR, eGFR and CAC and further supports the utility of these common clinical indices in risk stratification.

In this report, all predictor variables were standardized to allow for direct comparison of the relative importance of each in the context of risk for mortality. Based on calculated HR, UACR was the strongest predictor of all-cause mortality and CVD-mortality in the DHS after accounting for traditional CVD risk factors. Following additional adjustment for CAC, the HR suggested risk for both all-cause and CVD-mortality was greatest among those in the highest quartile of the measured UACR distribution. In the DHS, the upper quartile for serum creatinine concentration and UACR and the lower quartile for eGFR closely approximate accepted criteria for impairment of kidney function; UACR may perform differently in populations where renal function is better preserved. That said, UACR [25] and urine albumin excretion [14] have been reported to be stronger predictors of CVD-mortality than eGFR in subjects with T2D. Further, while proteinuria and eGRF were found to independently predict all-cause and CVD-mortality in the Taichung Diabetes Study, in this case investigators concluded that eGFR alone may be inadequate for risk prediction in T2D and that consideration of proteinuria can provide additional useful information [28]. The findings from the DHS further support that proteinuria must be considered as an important assessment of risk for adverse CVD outcomes in individuals with T2D and their family members.

### Limitations

It is unclear whether these findings are generalizable to other ethnic groups and non-diabetic individuals. The high rates of traditional CVD risk factors in DHS subjects represent an excessive burden of co-morbid conditions that contribute to risk for mortality. We attempted to account for many of these factors in the adjusted analyses. In addition, our assessment of kidney function and albuminuria was based on a single measure of eGFR and UACR; longitudinal monitoring may have provided a more accurate assessment of risk. Despite these limitations, the DHS represents a typical sample of community-dwelling EA T2D-affected individuals in the U.S. and provides a realistic assessment of the underlying CVD risk that is present in such populations.

## Conclusions

This study was an extension of our prior observations of independent relationships between albuminuria and CAC, and between CAC and mortality, and examined whether indices of kidney disease were predictive of mortality in EAs with T2D after considering the risk conferred by the presence of subclinical CVD. These results provide further support for the utility of routine clinical indices of kidney function and proteinuria in the prediction of mortality, independent from subclinical CVD and in the absence of more direct measures of CVD burden. As UACR is a modifiable risk factor and treatments exist for slowing the rate of eGFR decline, this information is clinically useful for risk stratification along with management of traditional CVD risk factors in EA T2D-affected individuals.

## Abbreviations

ACE: Angiotensin-converting enzyme; ARB: Angiotensin-receptor blocker; BMI: Body mass index; CAC: Coronary artery calcified plaque; CT: Computed tomography; CVD: Cardiovascular disease; DHS: Diabetes heart study; EAs: European Americans; eGFR: Estimated glomerular filtration rate; HbA1c: Glycosylated hemoglobin; HR: Hazard ratio; MDRD: Modification of diet in renal disease; ROC: Receiver operating characteristic; SD: Standard deviation; T2D: Type 2 diabetes mellitus; UACR: Urine albumin:creatinine ratio.

## Competing interests

The authors declare no conflicts of interest.

## Authors’ contributions

AJC collected mortality data, performed statistical analysis and prepared the manuscript; FCH assisted with statistical analysis and manuscript preparation; JJC was responsible for initial study design/sample ascertainment and reviewed the manuscript; BIF was responsible for initial study design/sample ascertainment and assisted with manuscript preparation; DWB was responsible for initial study design/sample ascertainment and assisted with manuscript preparation. All authors read and approved the final manuscript.

## Supplementary Material

Additional file 1 Cumulative incidence curves for all-cause mortality and CVD-mortality, results for T2D-only restricted analyses and area under the curve analysis for all-cause and CVD-mortality prediction. Click here for file
