# Supplementary material for: Glomerular filtration rate and albuminuria predict mortality independently from coronary artery calcified plaque in the Diabetes Heart Study
Source: Cardiovasc Diabetol. 2013 Apr 18;12:68. doi: 10.1186/1475-2840-12-68 (PMC3637614; doi:10.1186/1475-2840-12-68)
Supplement: Additional file 1 — Cumulative incidence curves for all-cause mortality and CVD-mortality, results for T2D-only restricted analyses and area under the curve analysis for all-cause and CVD-mortality prediction. [file 1475-2840-12-68-S1.pdf]

Additional Material

Figure 1

Cumulative incidence curves for (A) all-cause and (B) cardiovascular disease (CVD) mortality based on increasing serum albumin quartiles (Q1-Q4 scored in order of decreasing albumin concentrations).

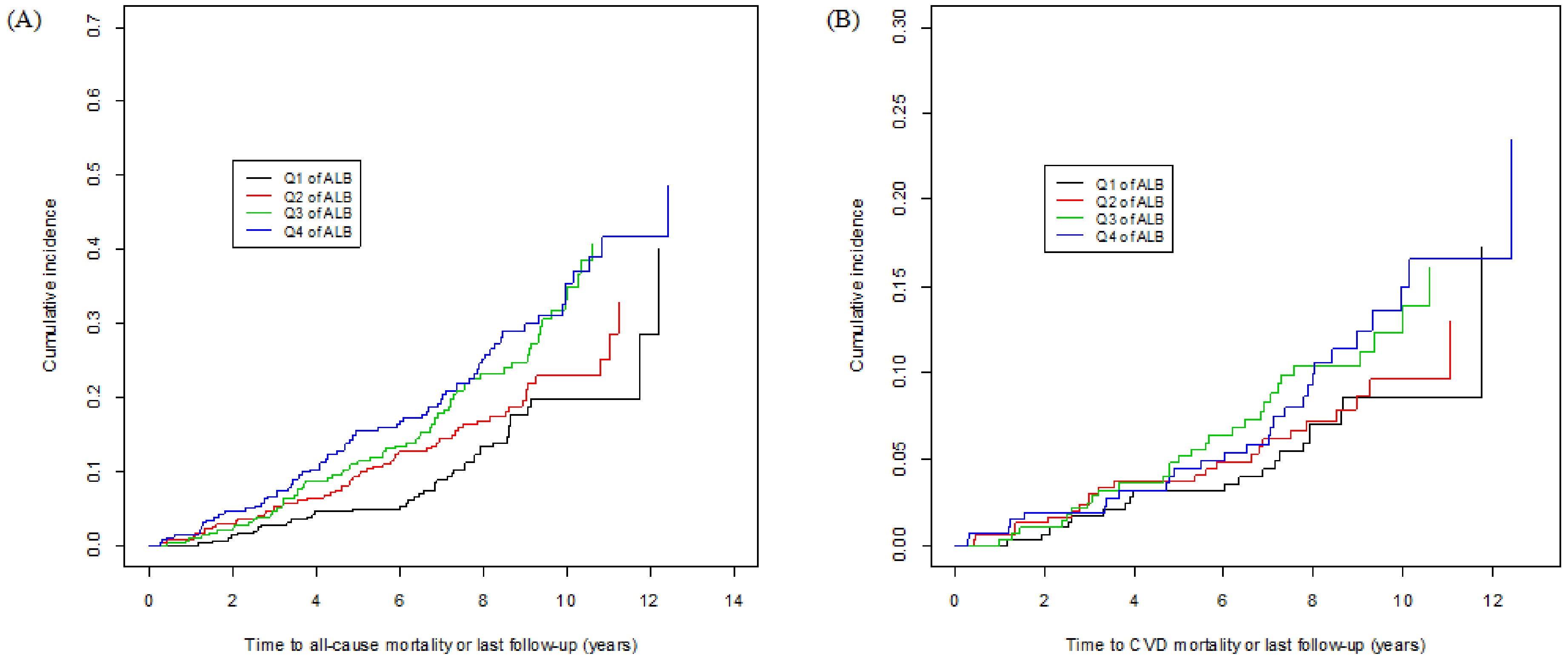

Figure 2

Cumulative incidence curves for (A) all-cause and (B) cardiovascular disease (CVD) mortality based on increasing serum creatinine quartiles (Q1-Q4, scored in order of increasing creatinine concentrations).

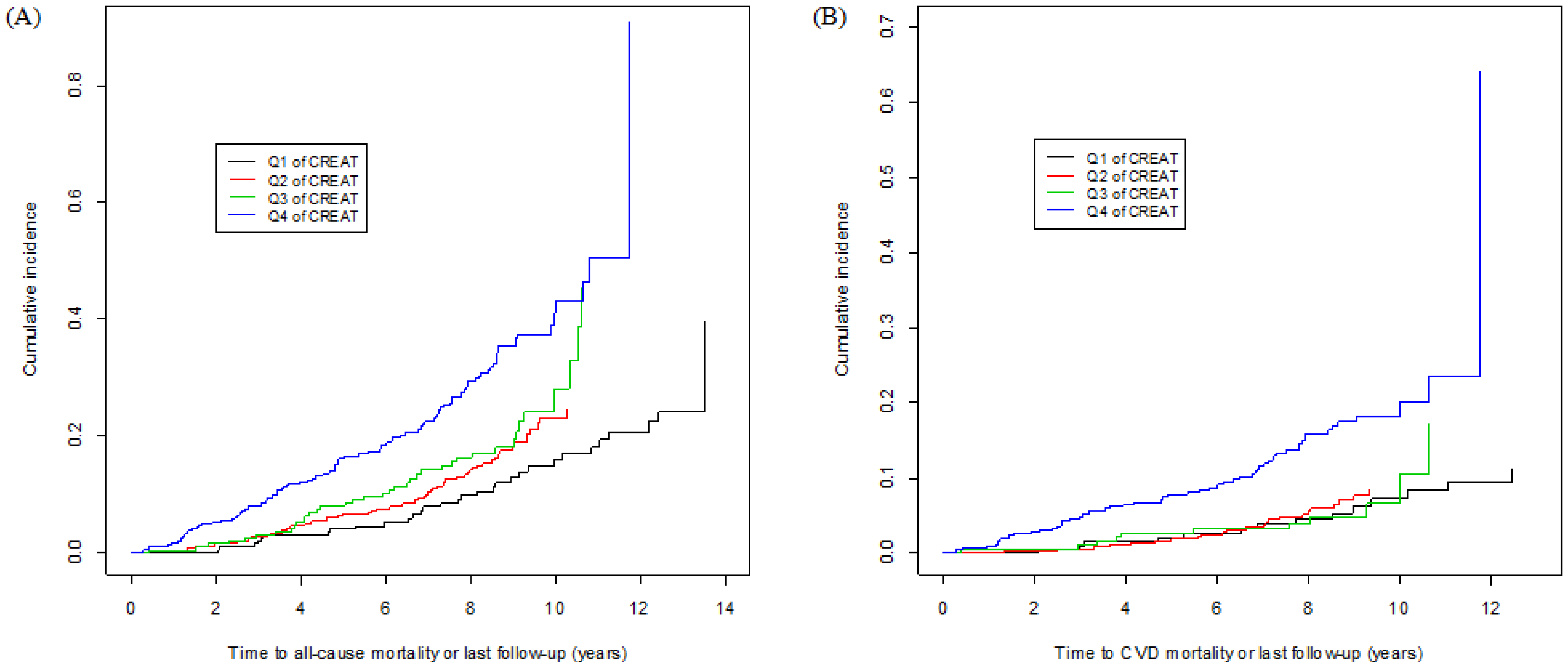

Figure 3

Cumulative incidence curves for (A) all-cause and (B) cardiovascular disease (CVD) mortality based on increasing urinary albumin:creatinine ratio (UACR) quartiles (Q1-Q4 scored in order of increasing UACR).

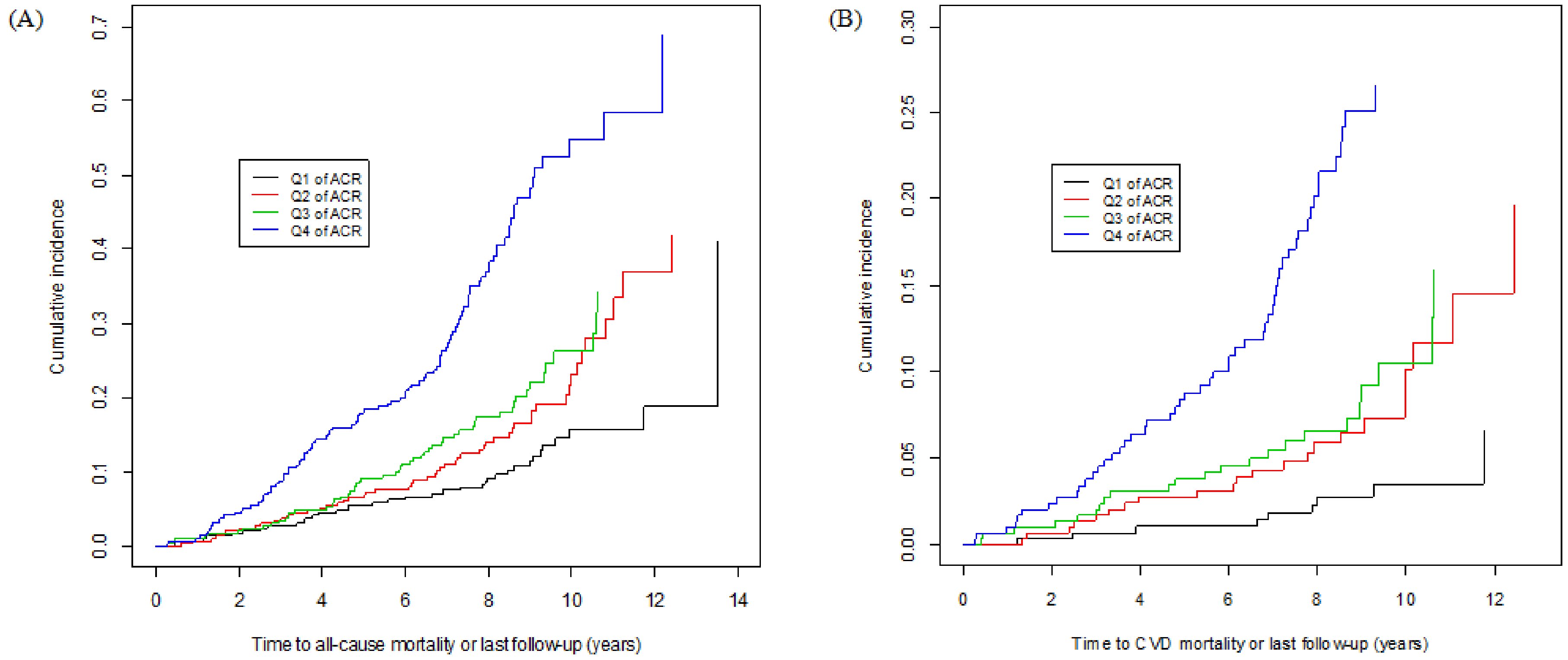

Figure 4

Cumulative incidence curves for (A) all-cause and (B) cardiovascular disease (CVD) mortality based on increasing estimated glomerular filtration rate (eGFR) quartiles (Q1-Q4 scored in order of deceasing eGFR).

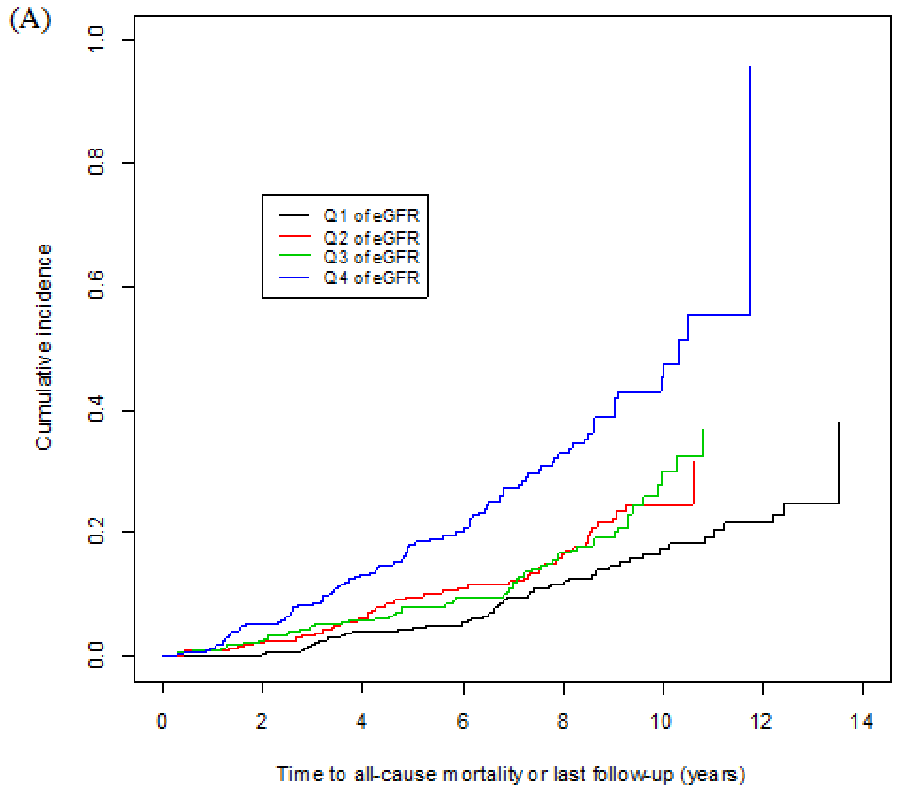

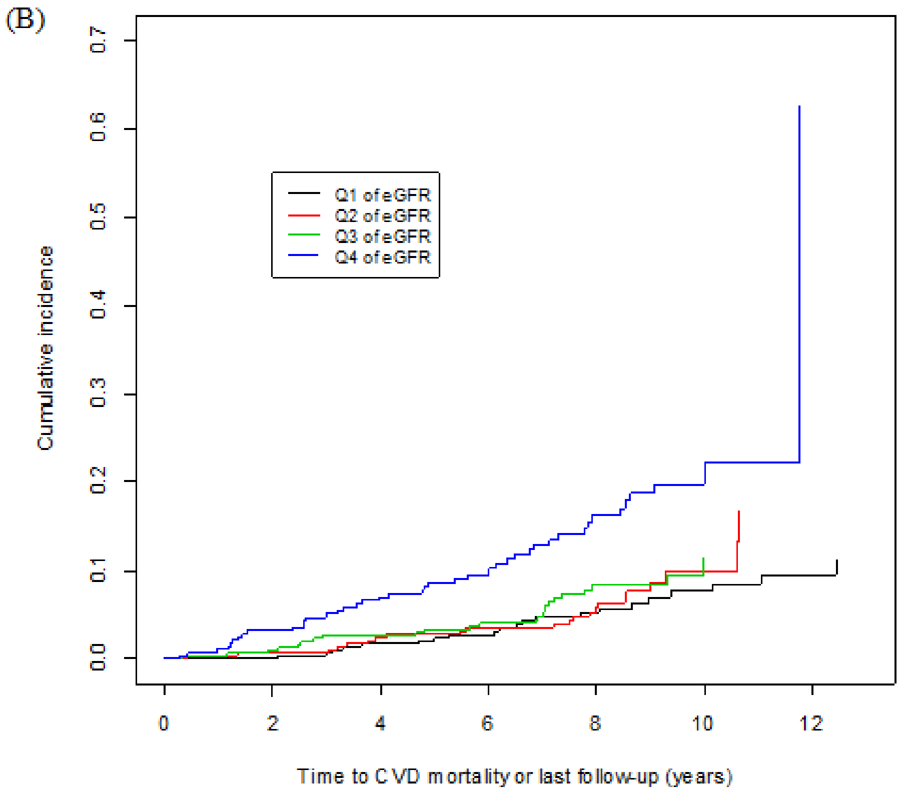

Table 1

Test for trend between ordinal measures of kidney disease and mortality in unadjusted models **for T2D affected individuals only.**

	All		T2D only	
	HR (CI)	p-value	HR (CI)	p-value
	**All-cause Mortality**			
Serum Albumin	0.82 (0.73-0.92)	0.0005	0.78 (0.69-0.88)	7.56×10^{-5}
Serum Creatinine	1.42 (1.26-1.60)	2.47×10^{-7}	1.39 (1.22-1.57)	2.94×10^{-7}
UACR	1.55 (1.37-1.74)	1.18×10^{-12}	1.47 (1.30-1.67)	1.26×10^{-9}
eGFR	0.71 (0.63-0.81)	1.07×10^{-7}	0.72 (0.64-0.82)	6.72×10^{-7}
	**CVD Mortality**			
Serum Albumin	0.85 (0.71-1.02)	0.09	0.82 (0.68-0.99)	0.04
Serum Creatinine	1.55 (1.28-1.88)	6.51×10^{-6}	1.51 (1.25-1.83)	2.92×10^{-5}
UACR	1.87 (1.55-2.26)	1.10×10^{-10}	1.85 (1.52-2.25)	6.75×10^{-10}
eGFR	0.69 (0.58-0.83)	5.86×10^{-5}	0.69 (0.57-0.83)	9.44×10^{-5}

Table 2

Association between continuous measures of kidney disease and mortality with covariate adjustment as indicated **for T2D affected individuals only.**

	Unadjusted		Partially Adjusted*		Fully Adjusted†		Fully Adjusted† + CAC	
	HR (CI)	p-value	HR (CI)	p-value	HR (CI)	p-value	HR (CI)	p-value
All-cause Mortality								
Serum Albumin	0.71 (0.62-0.81)	3.34×10^{-7}	0.69 (0.60-0.80)	3.48×10^{-7}	0.68 (0.59-0.79)	5.15×10^{-7}	0.68 (0.58-0.79)	1.19×10^{-6}
Serum Creatinine	1.53 (1.33-1.76)	1.67×10^{-9}	1.32 (1.13-1.55)	0.0007	1.28 (1.09-1.50)	0.003	1.29 (1.09-1.52)	0.003
UACR	1.52 (1.36-1.70)	1.66×10^{-13}	1.46 (1.29-1.65)	1.08×10^{-8}	1.39 (1.23-1.57)	1.79×10^{-7}	1.35 (1.19-1.53)	2.07×10^{-6}
eGFR	0.68 (0.60-0.78)	3.37×10^{-8}	0.76 (0.65-0.89)	0.0006	0.77 (0.66-0.90)	0.002	0.77 (0.65-0.91)	0.003
CVD Mortality								
Serum Albumin	0.79 (0.64-0.96)	0.02	0.78 (0.63-0.96)	0.02	0.75 (0.60-0.94)	0.01	0.77 (0.61-0.98)	0.03
Serum Creatinine	1.74 (1.40-2.15)	6.13×10^{-7}	1.55 (1.20-1.99)	0.0007	1.44 (1.13-1.85)	0.004	1.49 (1.15-1.92)	0.003
UACR	1.71 (1.47-1.99)	2.16×10^{-12}	1.63 (1.38-1.92)	1.33×10^{-9}	1.52 (1.28-1.80)	2.33×10^{-6}	1.47 (1.24-1.74)	1.03×10^{-5}
eGFR	0.63 (0.52-0.78)	1.95×10^{-5}	0.68 (0.54-0.86)	0.001	0.71 (0.56-0.90)	0.006	0.68 (0.53-0.89)	0.004

UACR=urine albumin:creatinine ratio; eGFR=estimated glomerular filtration rate.

*adjusted for age, sex, type 2 diabetes affection status, ACE/ARB medication use; † adjusted for age, sex, type 2 diabetes affection status, body mass index, current smoking, hypertension, dyslipidemia, ACE/ARB medication use, prior cardiovascular disease

Table 3

Association between categorical measures of kidney disease and all-cause mortality and cardiovascular disease (CVD) mortality **for T2D affected individuals only**.

			All-cause mortality		CVD-mortality	
	Quartiles	Quartlie Ranges	HR (95% CI)	p-value	HR (95% CI)	p-value
Serum	Q1-Q3	3.2-4.4				
Albumin†	Q4	4.4-5.3	1.70 (1.25-2.33)	0.0009	1.44 (0.89-2.35)	0.14
Serum	Q1-Q3	0.4-1.2				
Creatinine*	Q4	1.2-1.75	1.30 (0.94-1.80)	0.11	1.88 (1.16-3.05)	0.01
UACR*	Q1-Q3	0.5-44.5				
	Q4	44.5-4165.0	1.68 (1.25-2.65)	0.0005	2.52 (1.68-3.78)	7.95×10^{-6}
eGFR†	Q1-Q3	20.0-74.9				
	Q4	74.9-179.9	1.64 (1.19-2.27)	0.003	1.98 (1.24-3.16)	0.004

UACR=urine albumin:creatinine ratio; eGFR=estimated glomerular filtration rate.
Associations based on proportional hazards regression models adjusted for age, sex, type 2 diabetes affection status, body mass index, current smoking, hypertension, dyslipidemia, ACE/ARB medication use, prior CVD and coronary artery calcified plaque.
*For serum creatinine and UACR, quartiles were established based on increasing values across the distribution such that quartile 4 (Q4) captures the highest risk individuals. †For serum albumin and eGFR, quartiles were established based on decreasing values across the distribution such that Q4 again captures the highest risk individuals.

Table 4

Area under the curve (AUC) analysis for models predicting all-cause and cardiovascular disease (CVD) mortality.

Models	All-cause mortality		CVD-mortality	
	AUC	p-value	AUC	p-value
**Serum Albumin**				
Traditional	0.729	-	0.734	-
Traditional + Albumin	0.752	0.008*	0.747	0.15*
Traditional + CAC	0.762	0.001*	0.770	0.008*
Traditional + Albumin + CAC	0.781	0.02†	0.778	0.21†
**Serum Creatinine**				
Traditional	0.736	-	0.740	-
Traditional + Creatinine	0.734	0.75*	0.748	0.48*
Traditional + CAC	0.769	0.0008*	0.776	0.005*
Traditional + Creatinine + CAC	0.770	0.96†	0.784	0.40†
**eGFR**				
Traditional	0.736	-	0.740	-
Traditional + eGFR	0.734	0.694*	0.748	0.45*
Traditional + CAC	0.769	0.0008*	0.776	0.005*
Traditional + eGFR + CAC	0.770	0.87†	0.785	0.353†
**UACR**				
Traditional	0.738	-	0.737	-
Traditional + UACR	0.754	0.06*	0.767	0.03*
Traditional + CAC	0.771	0.001*	0.772	0.006*
Traditional + UACR + CAC	0.783	0.09†	0.795	0.06†

CAC = coronary artery calcified plaque; UACR=urine albumin:creatinine ratio; eGFR=estimated glomerular filtration rate. Models include traditional CVD risk factors with addition of a measure of kidney disease (either serum Albumin, serum Creatinine, eGFR or UACR), addition of CAC, or addition of both measures of kidney disease and CAC. *p-values are for comparisons to models containing traditional risk factors only; †p-values are for comparison to models containing traditional risk factors and CAC

Table 5

Association between measures of kidney function (quartiles 1-4; Q1-4) and all-cause mortality as assessed using proportional hazard regression with covariate adjustment as indicated.

		Unadjusted		Partially Adjusted*		Fully Adjusted†		Fully Adjusted† + CAC	
		HR (95%CI)	p-value	HR (95%CI)	p-value	HR (95%CI)	p-value	HR (95%CI)	p-value
Albumin	Q1 (Ref)	1.00	-	1.00	-	1.00	-	1.00	-
	Q2	1.25 (0.84-1.86)	0.28	1.35 (0.91-1.98)	0.13	1.33 (0.91-1.95)	0.14	1.27 (0.86-1.88)	0.22
	Q3	1.66 (1.13-2.44)	0.01	1.75 (1.20-2.56)	0.004	1.70 (1.15-2.50)	0.008	1.66 (1.12-2.17)	0.01
	Q4	1.81 (1.24-2.66)	0.002	1.93 (1.33-2.81)	0.0006	2.01 (1.38-2.93)	0.0003	2.03 (1.38-3.00)	0.0003
Creatinine	Q1 (Ref)	1.00	-	1.00	-	1.00	-	1.00	-
	Q2	1.34 (0.85-2.10)	0.20	1.21 (0.77-1.92)	0.41	1.20 (0.77-1.88)	0.43	1.38 (0.85-2.25)	0.20
	Q3	1.72 (1.04-2.82)	0.03	1.34 (0.81-2.24)	0.26	1.24 (0.74-2.06)	0.42	1.47 (0.84-2.57)	0.17
	Q4	2.77 (1.80-4.25)	2.72×10^{-6}	1.75 (1.07-2.86)	0.03	1.65 (1.01-2.70)	0.05	1.82 (1.07-3.11)	0.03
UACR	Q1 (Ref)	1.00	-	1.00	-	1.00	-	1.00	-
	Q2	1.62 (1.06-2.48)	0.02	1.55 (1.03-2.34)	0.04	1.42 (0.95-2.14)	0.09	1.59 (1.03-2.45)	0.04
	Q3	1.84 (1.21-2.80)	0.005	1.49 (0.98-2.27)	0.07	1.36 (0.89-2.09)	0.15	1.55 (0.99-2.43)	0.06
	Q4	3.82 (2.60-5.63)	1.05×10^{-11}	2.89 (1.95-4.28)	1.34×10^{-7}	2.44 (1.64-3.63)	1.01×10^{-5}	2.53 (1.68-3.82)	8.98×10^{-6}

eGFR	Q1 (Ref)	1.00	-	1.00	-	1.00	-	1.00	-
	Q2	1.51 (1.02-2.22)	0.04	1.38 (0.94-2.02)	0.09	1.36 (0.93-2.00)	0.11	1.58 (1.05-2.38)	0.03
	Q3	1.53 (1.02-2.28)	0.04	1.24 (0.83-1.85)	0.25	1.17 (0.78-1.77)	0.45	1.26 (0.82-1.96)	0.30
	Q4	2.92 (1.99-4.27)	2.31×10^{-8}	2.03 (1.35-3.04)	0.0007	1.85 (1.22-2.82)	0.004	2.02 (1.30-3.16)	0.002

UACR=urine albumin:creatinine ratio; eGFR=estimated glomerular filtration rate.

*adjusted for age, sex, type 2 diabetes affection status, ACE/ARB medication use; † adjusted for age, sex, type 2 diabetes affection status, body mass index, current smoking, hypertension, dyslipidemia, ACE/ARB medication use, prior cardiovascular disease

Table 6

Association between measures of kidney function (quartiles 1-4; Q1-4) and all-cause mortality as assessed using proportional hazard regression with covariate adjustment as indicated.

		**Unadjusted**		**Partially Adjusted***		**Fully Adjusted†**		**Fully Adjusted† + CAC**	
		**HR (95%CI)**	**p-value**	**HR (95%CI)**	**p-value**	**HR (95%CI)**	**p-value**	**HR (95%CI)**	**p-value**
Albumin	Q1 (Ref)	1.00	-	1.00	-	1.00	-	1.00	-
	Q2	1.08 (0.59-1.98)	0.80	1.19 (0.66-2.14)	0.56	1.16 (0.64-2.07)	0.63	1.19 (0.66-2.12)	0.56
	Q3	1.47 (0.83-2.61)	0.19	1.56 (0.88-2.75)	0.13	1.65 (0.92-2.94)	0.09	1.57 (0892-2.79)	0.12
	Q4	1.53 (0.85-2.76)	0.16	1.64 (0.91-2.96)	0.10	1.76 (0.97-3.18)	0.06	1.69 (0.93-3.10)	0.09
Creatinine	Q1 (Ref)	1.00	-	1.00	-	1.00	-	1.00	-
	Q2	1.09 (0.56-2.12)	0.80	0.95 (0.48-1.87)	0.88	0.96 (0.49-1.87)	0.89	1.01 (0.50-2.04)	0.98
	Q3	1.09 (0.49-2.42)	0.83	0.86 (0.39-1.89)	0.86	0.76 (0.34-1.66)	0.49	0.85 (0.38-1.90)	0.69
	Q4	3.03 (1.68-5.47)	0.0002	2.02 (1.01-4.02)	0.05	1.83 (0.91-3.72)	0.09	1.92 (0.92-4.00)	0.08
UACR	Q1 (Ref)	1.00	-	1.00	-	1.00	-	1.00	-
	Q2	2.68 (1.27-5.65)	0.01	2.62 (1.25-5.50)	0.01	2.44 (1.17-5.08)	0.02	2.59 (1.18-5.68)	0.02
	Q3	3.01 (1.41-6.42)	0.004	2.45 (1.13-5.31)	0.02	2.27 (1.05-4.94)	0.04	2.43 (1.09-5.42)	0.03
	Q4	7.67 (3.84-15.31)	7.64×10^{-9}	5.73 (2.81-11.68)	1.57×10^{-6}	4.72 (2.29-9.73)	2.58×10^{-5}	4.84 (2.35-9.99)	1.97×10^{-5}

eGFR	Q1 (Ref)	1.00	-	1.00	-	1.00	-	1.00	-
	Q2	1.29 (0.72-2.28)	0.39	1.18 (0.67-2.10)	0.57	1.11 (0.62-1.99)	0.74	1.20 (0.64-2.23)	0.57
	Q3	1.37 (0.78-2.43)	0.27	1.16 (0.64-2.11)	0.62	1.02 (0.55-1.90)	0.95	1.03 (0.54-1.95)	0.94
	Q4	3.01 (1.18-5.10)	4.05×10^{-5}	2.34 (1.29-4.25)	0.005	2.00 (1.09-3.69)	0.03	2.07 (1.10-3.90)	0.02

UACR=urine albumin:creatinine ratio; eGFR=estimated glomerular filtration rate.

*adjusted for age, sex, type 2 diabetes affection status, ACE/ARB medication use; † adjusted for age, sex, type 2 diabetes affection status, body mass index, current smoking, hypertension, dyslipidemia, ACE/ARB medication use, prior cardiovascular disease
